# Group cognitive remediation therapy for children and adolescents in intensive day-patient treatment for anorexia nervosa: a feasibility study

**DOI:** 10.1007/s40211-022-00420-5

**Published:** 2022-06-15

**Authors:** Charlotte Rhind, Mishka Mahdi, Mima Simic, Jonathan Espie, Kate Tchanturia

**Affiliations:** 1 South London and Maudsley NHS Foundation Trust, London, UK; 2 Department of Clinical, Educational and Health Psychology, University College London, London, UK; 3 Department of Psychological Medicine, Institute of Psychiatry, Psychology and Neuroscience, King’s College London, De Crespigny Park, PO59, SE5 8AF London, UK; 4 Psychology Department, Illia State University, Tbilisi, Georgia

**Keywords:** Anorexia nervosa, Cognitive remediation, Feasibility study, Neuropsychological assessment, Autism Spectrum Disorder, Anorexia nervosa, Kognitive Remediationstherapie, Machbarkeitsstudie/Projektstudie, Neuropsychologische Beurteilung, Autismus-Spektrum-Störung (ASS)

## Abstract

**Background:**

Cognitive Remediation Therapy (CRT) is an adjunct treatment targeting set-shifting (SS) and weak central coherence (CC) thought to play a role in maintaining anorexia nervosa (AN). This study aimed to test the feasibility of brief group CRT for young people with AN in a day-patient setting using neuropsychological assessment of SS and CC. It also explored the benefit for young people with Autism Spectrum Disorder (ASD) features.

**Methods:**

Twenty female day-patients (12–18 years) with AN or Atypical AN completed neuropsychological assessment of SS (using the Wisconsin Card Sorting Task and the Brixton Spatial Anticipation Test) and of CC (using the Rey Osterrieth Complex Figures Task) pre and post 4 sessions of group CRT. Baseline ASD features and weight change pre and post were measured. Feasibility was assessed regarding the recruitment process, intervention feedback, suitability of neuropsychological assessment measures, and effect sizes.

**Results:**

Study uptake was 95%, intervention retention was 91%, and group CRT was rated as acceptable. Medium to large effect size improvements were found across measures of SS (*d*_*z*_ = 0.44 to *d*_*z*_ = 0.90) and medium size improvements found in CC (*d*_*z*_ = 0.59). Young people with ASD features showed smaller to similar size improvements in SS and larger improvements in CC. There was a moderate (not significant) correlation with level of weight restoration.

**Conclusions:**

Important study parameters have been estimated in order to plan a future definitive trial of brief group CRT in a day-patient setting using neuropsychological assessment. These findings also have implications for the use of brief group CRT in young people with ASD features.

## Abstract (de)

**Hintergrund:**

Die kognitive Remediationstherapie (CRT) ist eine begleitende Behandlung einer Verschiebung von Sets („set shifting“, SS) und einer schwachen zentralen Kohärenz (CC), von denen angenommen wird, dass sie eine Rolle bei der Aufrechterhaltung der Anorexia nervosa (AN) spielen. Ziel dieser Studie war es, die Durchführbarkeit einer kurzen CRT-Gruppenbehandlung für junge Menschen mit AN in einer Tagesklinik zu testen, wobei eine neuropsychologische Beurteilung von SS und CC vorgenommen wurde. Es wurde auch der Nutzen für junge Menschen mit Autismus-Spektrum-Störung (ASD) untersucht.

**Methoden:**

Zwanzig weibliche teilstationäre Patienten (12–18 Jahre) mit AN oder atypischer AN erhielten eine neuropsychologische Beurteilung zu SS (unter Verwendung der Wisconsin Card Sorting Task und des Brixton Spatial Anticipation Tests) und zu CC (mittels des Rey Osterrieth Complex Figures Task) vor und nach 4 Sitzungen der Gruppen-CRT. Die ASD-Basismerkmale und die Gewichtsveränderung vor und nach der Behandlung wurden gemessen. Die Durchführbarkeit wurde im Hinblick auf den Rekrutierungsprozess, das Interventionsfeedback, die Eignung der neuropsychologischen Beurteilungsmaßnahmen und die Effektgrößen bewertet.

**Ergebnisse:**

Die Studie wurde zu 95 % begonnen, die Beibehaltung der Intervention lag bei 91 %, und die CRT-Gruppe wurde als akzeptabel eingestuft. Es wurden mittelgroße bis große Verbesserungen bei allen Messgrößen für SS (*d*_*z*_ = 0,44 bis *d*_*z*_ = 0,90) und mittelgroße Verbesserungen bei CC (*d*_*z*_ = 0,59) festgestellt. Junge Menschen mit ASD-Merkmalen zeigten kleinere bis ähnlich große Verbesserungen in SS und größere Verbesserungen in CC. Es bestand eine mäßige (nicht signifikante) Korrelation mit dem Grad der Wiederherstellung des Gewichts.

**Schlussfolgerungen:**

Es wurden wichtige Studienparameter geschätzt, um eine künftige definitive Studie über kurze Gruppen-CRT in einer teilstationären Einrichtung mit neuropsychologischer Beurteilung zu planen. Diese Ergebnisse haben auch Auswirkungen auf den Einsatz von kurzen Gruppen-CRT bei jungen Menschen mit ASD-Merkmalen.

## Introduction

Anorexia Nervosa (AN) is a severe illness with onset typically during mid-adolescence [1]. The aetiology of AN is complex and multifactorial. Risk and maintaining factors can be hard to disentangle, including the role of heritability and biological factors, neuropsychological profile, socio-emotional profile, and socio-cultural factors [2]. Prevalence data highlights a need for effective and early treatment that targets factors contributing to illness maintenance and progression [3].

The neuropsychological profile is a key challenge associated with the treatment of AN. Individuals exhibit inefficiencies in cognitive processing that may compromise the efficacy of talking therapies [4]. In addition to perfectionist and obsessive-compulsive features, the cognitive functioning component of the model outlines a neuropsychological profile in AN characterised by poor set-shifting (SS) and difficulties with global processing. Poor SS may manifest as rigid approaches to problem solving and perseverance of maladaptive thinking and behavioural patterns [5], and weak central coherence (CC) as excessive preoccupation with detail at the expense of the ‘bigger picture’ [6]. In relation to AN, these may relate to a pathological fixation with calories and fat content, weight gain and loss, exercise routines, and other rule-bound practises [7].

Small-to-medium effect size difficulties have been found in SS in adults with AN relative to healthy controls [8], and small effect size inefficiencies in CC using the Rey Osterrieth Complex Figures Task (ROCFT) [6]. Results have been less conclusive in the child and adolescent literature. A systematic review in young people with AN found similar inefficient CC as found in adults with AN using the ROCFT [7], but children and adolescents with AN were non-significant compared to healthy controls in SS [9]. The findings from a large study which employed more robust neuropsychological tasks found significant differences between young people with AN and healthy control groups with medium effect sizes (*d* = 0.49) on the Wisconsin Card Sorting Test (WCST) and ROCFT (*d* = 0.57) [10]. Findings of cognitive inefficiencies occurred independent of clinical variables, most notably, weight status indicates that they may relate to underlying traits rather than explained fully by the secondary effects of starvation on the brain.

Empirical evidence for CRT’s effectiveness has been growing, a systematic review concluded that CRT appears to be associated with small to medium effect size improvements in SS and in CC [11]. Smaller effect size improvements have been found for subgroups of adults with AN with high scores on ASD measure [12], suggesting that CRT may be require adaptation for this subgroup, however this study was limited to self-report measures of SS and CC. Less is known about treatment response in ASD, however as ASD symptoms are over-represented in AN [13] further research is warranted. A systematic review and meta-analysis evaluated CRT in young people with AN and suggested small effect size improvements in CC using the ROCFT (*d* = 0.41) but no significant improvements in SS using the Trail Making Task (*d* = 0.03) [14]. The authors highlighted issues of quality across studies and concluded that it is difficult to establish whether CRT is truly less effective in adolescents relative to adults, or whether effect sizes in young people studies would improve with more robust study designs.

Group CRT is acceptable to children and adolescents with AN [15, 16] and has shown promise in improving self-reported cognitive flexibility and ‘bigger picture’ thinking as well as improving motivation to change [17]. However, little is known about the benefits of group CRT in young people with AN when employing a more robust design similar to those used in adult studies [11]. Furthermore, there is inconsistency across studies in selection of the most suitable outcome measures. In planning a larger trial, an important methodological consideration is study recruitment and attrition which is a challenge in AN studies.

The current study aimed to test the feasibility of evaluating group CRT in a day-patient treatment programme for young people with AN using neuropsychological measures of both SS and CC in order to establish important study parameters needed to design a definite trial in the future. This included establishing recruitment rate, variance estimate of CRT outcome measures which can be used for future power calculations, and characteristics of the CRT outcome measures. A second aim was to perform exploratory preliminary analysis to explore whether improvements in SS and CC may relate to changes in weight restoration and may have smaller effect sizes for those scoring high on ASD measures or with an ASD diagnosis.

We hypothesized that 1) It would be feasible to recruit a minimum of 20 participants over the planned 10 month recruitment phase from the day-patient programme and to complete an assessment at Time 1 (prior to starting group CRT) and Time 2 (after 4th session); 2) Effect sizes using pre and post measures for participants receiving group CRT (in addition to standard treatment) would demonstrate positive change (improvements in SS, CC); and 3) Neuropsychological measures employed in the current study sample would be suitable for use in evaluating group CRT. It was hypothesized that improvements post-intervention may relate to changes in weight restoration, and ASD features (defined by having a diagnosis of ASD or scoring high on ASD measures).

## Methods

This feasibility study used a pre and post uncontrolled case series design.

### Participants

Twenty-two young people were recruited from the South London and Maudsley NHS Foundation Trust (SLaM) specialist CAMHS eating disorders day-patient programme (the Intensive Treatment Program ‘ITP’). Participants could participate if they were: 1) aged 10–18 years, 2) receiving treatment in the ITP with a primary diagnosis of AN or atypical AN using DSM‑5 and ICD-10 criteria, derived by a 3-hour multi-disciplinary and multi-informant clinical interview on intake to the programme, and 3) planning attendance on the day of the group. Exclusion criteria were English non-fluency, current or recent involvement in another research study, and severe diagnosed comorbidity at intake (e.g. psychosis, severe learning disability).

### Procedure

Young people provided consent (if 16 or older) or assent with parental/guardian consent. Participants were invited to complete two assessment sessions with CR that included questionnaires and experimental assessment at Time 1 (prior to the first group CRT session) and Time 2 (after completing the 4th CRT session). Treatment was not changed in any way while taking part in the study; they continued to receive their standard treatment (other group therapies, FT-AN, individual therapy, supervised meals and multi-family therapy groups for some young people). Due to the current study being a pragmatic feasibility study, participants were included even if they had already completed some CRT sessions; in these cases, the number of sessions completed prior to the study were logged, and the Time 2 assessment was conducted following the 4th group CRT session since the Time 1 assessment. Four CRT sessions have been used in previous studies as a minimum completion [5, 16], and therefore a minimum of 4 sessions was used for the purposes of completing a pre and post intervention assessment. CRT was primarily delivered in group format, however, up to 2 individual sessions were made available where necessary when the minimum dose of group CRT was not achieved due to practical reasons (e.g. participant transitioning back to school). Amazon vouchers (£ 10) were offered upon completion of Time 2 measures and all participants were provided a debrief regarding the study aims.

### The CRT group

Data was collected from four cycles of the ITP CRT group. The group followed a manualised 8‑session programme [18] that was developed in the service and is widely available (http://media.wix.com/ugd/2e1018_bb804c6eeca3421d98e4fb29f20dea1e.pdf). It was co-facilitated by the lead researcher CR and an Assistant Psychologist, both having completed CRT training with co-author KT. Weekly sessions lasted 45 min. Format was consistent including icebreakers, homework review, 2–3 practical ‘experimental’ exercises followed by discussion using every day examples integrating psych-education about the brain, cognitive styles in AN, why flexible and bigger picture thinking is helpful in recovery and planning homework with facilitators [19]. Facilitators took a motivational and collaborative stance and took part in the group exercises. Sessions 1–3 and 5–7 practise thinking styles: bigger picture thinking, flexible thinking, and multi-tasking, and sessions 4 and 8 are summary sessions to consolidate learning.

### Measures

Demographic and clinical information were collated from clinical records at Time 1 (see Table 1). Data on intellectual functioning using Wide Range Achievement Test, Fourth Edition [20] was collated at Time 1 because SS and CC is closely related to intellectual or achievement ability levels [21]. Data on BMI and expected body weight percentage; %EBW was collated from records at Time 1 and 2.

**Table 1:** Sample characteristics at baseline (counts (%) for categorical and *M* *(SD) *for continuous variables)

Variable	*N* = 20, Frequency (%)/M (SD)
*Age*	15.40 (1.54)
*Female*	20 (100%)
*Ethnicity*	
White British	17 (85%)
British Other	1 (5%)
White and Black Caribbean	1 (5%)
Indian/British Indian	1 (5%)
*WRAT‑4 (Wide Range Achievement Test 4) Reading*^*a*^	108.15 (30.45)
*WRAT‑4 Spelling*^*a*^	101.60 (33.43)
*WRAT‑4 Maths*^*a*^	94.75 (31.74)
*Primary Diagnosis*	
AN (Anorexia Nervosa)	18 (90%)
Atypical AN	2 (10%)
*%Expected Body Weight*	82.31% (6.73)
*Body Mass Index*	16.69 (1.32)
*Duration of illness (months)*	22.15 (18.54)
*Age of illness onset*	13.60 (1.67)
*No. of patients with previous hospitalisations*	10 (50%)
*Type of admission*	
Paediatric ward/Accident & Emergency (A&E)	5 (25%)
Eating Disorder Unit	5 (25%)
*No. of patients with a psychiatric comorbidity*	7 (35%)
*Comorbidity type and Autism Spectrum Disorder (ASD)*^*b*^	
Depressive Disorders	4 (20%)
Anxiety Disorders	4 (20%)
Obsessive Compulsive Disorder (OCD)	3 (15%)
ASD	2 (10%)
*No. of patients receiving psychotropic medication*	15 (75%)
*Type of medication*	
Olanzapine	8 (40%)
Sertraline	6 (30%)
Fluoxetine	3 (15%)
Citalopram	2 (10%)
Quetiapine	1 (5%)
Gabapentin	1 (5%)
Escitalopram	1 (5%)
*Social Communication Questionnaire*	7.53 (5.11)

*%EBW* percentage of estimated body weight, *P’s* participants

^a^WRAT‑4 standard score is reported.

^b^Includes multiple diagnoses

The Eating Disorder Examination Questionnaire (EDE-Q) [22] (validated for use in young people) is a standardized self-report questionnaire measuring severity of the characteristic psychopathology of EDs. Community norms for young people for global score are 1.6 (SD = 1.4) and a global score ≥ 3 is indicative of an ED [22]. For internal consistency there are different reports about the acceptable values of alpha, usually using alpha coefficient > 0.80 as acceptable [23]. Cronbach’s α in the current study at Time 1 and Time 2 was 0.93 and 0.57, respectively.

The Motivational Ruler used at Time 1 and 2 is a self-report measure of importance and confidence to change using Visual Analogue Scale (0–10). Higher scores reflect more motivation for ED recovery.

Revised Child and Anxiety and Depression Scale (RCADS) [24] is a standardised self-report measure of anxiety and depression symptoms that has been validated for use in young people. Scores of 70 and above are considered the clinical range. Cronbach’s α in the current study at Time 1 and Time 2 was 0.95 and 0.94, respectively.

The Social Communications Questionnaire (SCQ 20-item) [25], lifetime version, is a standardised measure of ASD traits over the developmental trajectory and was completed by parents as informants at Time 1. Scores above 15 are used as suggestive of clinically relevant social and communication difficulties. Cronbach’s α in the current study was 0.88.

The Social Responsiveness Scale (SRS-2) [26] is a standardised measure assessing severity of current levels of social communication impairment and was completed by parents as informants. This measure includes norms based on both males and females, enabling a potentially more sensitive assessment of ASD features as they likely manifest in females compared to diagnostic manuals and related measures based predominantly on male norms. T scores of 59 and below are considered within the normal limits and scores 66 to 75 within the moderate range and 76 or higher severe range typically found in children with a diagnosis of ASD. Cronbach’s α in the current study at Time 1 and Time 2 was 0.96 and 0.96, respectively.

The patients’ scores on ASD measures were divided in to relative ‘low’ and ‘high’ groups according to the clinical cut-off scores for both measures (using the more conservative cut-off on the SRS‑2 for moderate range difficulties) and according to an existing ASD diagnosis. Relative ‘high’ ASD features was assigned when participants had either a pre-existing diagnosis of ASD or scored above the clinical cut-offs on either measure in line with the methodology used in previous similar CRT research exploring the benefit for those with possible ASD features [12].

The Detail and Flexibility questionnaire (D-FLEX), a self-report scale consisting of 24 items assessing cognitive rigidity and attention to detail [27]. The clinical cut-off for the cognitive rigidity subscale is 53 and above, and 44 for the attention to detail subscale. Although it has only been validated in adults with AN, the scale has displayed high internal reliability and construct validity in both subscales D‑FLEX [27]. Cronbach’s alpha in the current sample at Time 1 and Time 2 was 0.93 and 0.93, respectively.

#### Neuropsychological assessment measures

Set-Shifting: The WCST version 4 [28] was administered at Time 1 and 2. Participants are required to match a number of stimulus cards to one of four category cards. Cards can be matched by colour, number or shape, and the rule must be worked out by trial and error based upon the feedback received. Once the participant has correctly matched the card for 10 consecutive sorts, the sorting rule changes and the participant must shift their response to work out the new sorting rule. The rule changes up to five times throughout the task, and every time a participant correctly completes a sort this is termed ‘completing a set’. The most commonly reported outcome is the number of perseverative errors made by the participant, with scores on this variable used as an indicator of levels of cognitive flexibility (SS). In order to mimic the largest dataset in adult AN [5], the current study also reported measures of general performance, perseveration, conceptual ability and response consistency. For further descriptions of each of these measures see Tchanturia and colleagues [5]. The present study replicated test parameters of recent studies [5, 10] whereby computerised visual and audio feedback was given in a male voice one second after each sort was been made.

The Brixton Spatial Anticipation Test [29] assessing SS was administered at Time 1 and Time 2. It is a concept (or ‘rule’) attainment task, which incorporates switching between mental representations. The test consists of 56 trials and each has the same array of ten circles in a two by five matrix. On each trial, one circle is filled in with the colour blue. The position of this changes from trial to trial, with the participant having to determine a rule that governs the sequence of changes, predicting the location of the filled circle for the next trial. As the test progresses, the rule changes, requiring detection of the new rule. The total number of errors made on the test can be used to construct a scaled score. Consistent with use of the Brixton Test in other studies in AN, the computerised version of the Brixton Test was used at Time 1 and a different but matched version (i.e. different order of stimulus/rules) used at Time 2 in order to reduce the impact of practise effects from repeated measurement—albeit presenting different test stimulus does not fully remove this effect. The outcome measure used was the number of errors made (maximum number is 54), with higher scores reflecting more perseverative errors (i.e. poorer SS) similar to the WCST.

Central Coherence: The ROCFT [30], a pen and paper task measuring global processing ability and CC, was administered at Time 1 and 2. Participants are required to accurately copy a complex figure and the drawing strategy adopted by the participant is measured. It was scored according to a modification of Booth’s scoring method [31], which incorporates both the order in which the participant chooses to draw the elements (whether preference is shown to global or detailed elements) and the style in which they are drawn in (fragmented or coherently), with a modification applied as in other similar CRT studies aimed to reduce ceiling effects. Order index (OI) and style indexes (SI) are computed and are added to give the CC Index (CCI). For more details see Lang and colleagues [10].

The Group Satisfaction Questionnaire was completed as self-report by participants at Time 2 within an anonymized questionnaire pack. This measure included four questions asking participants to rate areas of treatment satisfaction (enjoyment, usefulness, skills and strategies acquired, and session length) rated using a 5-point likert scale (higher scores reflecting more satisfaction). This measure was adapted to include an ‘open feedback’ section, to enable participants to expand on their thoughts and feedback about the group CRT intervention.

## Results

**Fig. 1:**
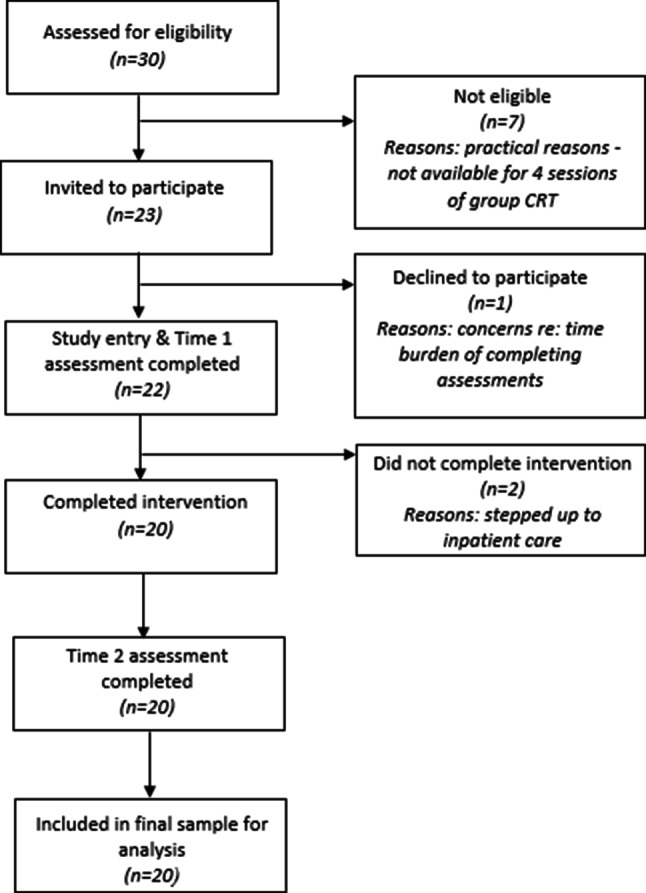
Study flow chart

All 20 participants were female. Mean age was 15 (SD = 1.54) with range 12 to 17 years. Recruitment averaged 3 participants per month. Demographic and clinical characteristic at baseline are presented in Table 1. See Fig. 1 for study flow chart and reasons for exclusion. No risk or safeguarding issues or serious incidents arose.

### Primary outcomes

One outlier was detected at Time 1 and Time 2 on the WCST, using visual inspection of boxplot and assessing for standardised residual values above or below the recommended cut-off of 0.3 (values of 3.2 and 3.9 at Time 1 and Time 2). WCST responses for this case were removed from analysis. There were no outliers using the Brixton Test or the ROCFT. Effect sizes for the WCST between time points was calculated using Cohen’s d both with and without the outlier. Table 2 shows the change score, effect size and 95% confidence intervals from Time 1 (pre-CRT) to time 2 (following 4th session of group CRT) on the main outcome measures. Log transformation was used for the WCST main outcome variable (% perseverative errors) due to positive skew in the data. Log or square root transformations did not improve the error distribution for data on the ROCFT, and so raw data was used in analysis.

**Table 2:** Mean difference pre-to-post group CRT in neuropsychological measures

Variable	Time 1 score *M* (*SD*)	Time 2 score *M* (*SD*)	Change score^a^ *M* (*SD*)	Effect size Cohen’s *d*_*z*_ (95% CI)
**SS outcomes: Wisconsin Card Sorting Task (*n*** **=** **19)**				
*General performance measures*				
Number of trials administered	89.84 (18.37)	84.05 (17.73)	–	–
Total correct responses	68.58 (9.19)	69.26 (7.85)	–	–
Total response errors	21.26 (19.98)	14.79 (10.89)	–	–
Total response errors %	21.26 (13.75)	16.16 (8.03)	–	–
Total categories completed	5.58 (1.26)	5.79 (0.92)	–	–
*Perseveration measures*				
Perseverative responses	10.32 (7.65)	7.63 (4.87)	–	–
Perseverative responses %	10.63 (5.48)	8.42 (3.56)	–	–
Perseverative errors	9.58 (6.56)	7.37 (4.40)	–	–
**Perseverative errors %**^b^	**10.00 (4.62)**	**8.16 (3.06)**	**1.84 (3.45)**	**0.53 (0.18, 3.51)**
**Perseverative errors % (transformed)**^c^	**0.96 (0.17)**	**0.89 (0.15)**	**0.08 (0.15)**	**0.53 (0.01, 0.15)**
Non-perseverative errors	11.68 (15.21)	7.42 (6.95)	–	–
Non-perseverative errors %	11.32 (11.18)	8.05 (5.53)	–	–
*Conceptual ability measures*				
SS outcomes: Wisconsin Card SortingTask (*n* = 19)				
Trials to complete first category	16.84 (19.48)	13.26 (8.37)	–	–
Conceptual level responses	63.53 (12.95)	66.84 (6.30)	–	–
Conceptual level responses %	73.84 (18.63)	81.53 (11.07)	–	–
*Response consistency measures*				
Failure to maintain set	0.37 (0.68)	0.47 (1.02)	–	–
Learning to learn	−2.45 (8.42)	−0.72 (1.63)	–	–
**SS outcomes: Brixton Test (*n*** **=** **20)**				
**Brixton Test Number of Errors**^**d**^	**8.00 (3.57)**	**5.30 (2.27)**	**2.70 (2.47)**	**1.09 (1.54, 3.86)**
Brixton Test Scaled Score	8.90 (1.41)	9.75 (0.55)	–	–
**CC outcomes: Rey Osterrieth Complex Figures Test (*n*** **=** **20)**				
ROCFT Order Index	1.90 (0.74)	2.29 (0.61)	–	–
ROCFT Style Index	1.54 (0.38)	1.75 (0.31)	–	–
**ROCFT—CC Index**^e^	**1.38 (0.39)**	**1.59 (0.32)**	**0.21 (0.30)**	**0.70 (0.07, 0.34)**

*SS* Set-shifting, *CC* Central Coherence, *d*_*z*_ Standardised mean change with change score standardisation

^a^Change score calculated in the direction of expected improvement (i.e. positive value change score will reflect improvement in this variable at Time 2, and negative change score will reflect worsening of that variable at Time 2)

^b^Index score on WCST (one outlier removed)

^c^Index score on WCST transformed (log transform, one outlier removed)

^d^Index score on the Brixton test

^e^Index score on the ROCFT

There was a medium effect size improvement on the WCST (ES = 0.53, 95% CI = 0.18, 3.51, and for the T score 95% CI = 0.01, 0.15), a large effect size improvement on the Brixton test (ES = 1.09, 95% CI = 1.54, 3.86) and a medium effect size improvement on the ROCFT (ES = 0.70, 95% CI = 0.07, 0.34) (Table 3).

**Table 3:** Clinical variables at time 1 and time 2 (*M,* *SD*)

Variable	*N*	Time 1 score *M* (*SD*)	*N*	Time 2 score *M* (*SD*)	Change score^a^	Effect Size *d*_*z*_ (95% CI)
%EBW	20	82.31 (6.73)	20	86.54 (8.35)	4.23 (3.60)	1.18 (2.54, 5.91)
BMI	20	16.69 (1.32)	20	17.61 (1.52)	0.92 (0.70)	1.31 (0.59, 1.24)
EDE‑Q Restraint	15	3.73 (1.69)	16	3.16 (2.05)	0.86 (1.48)	0.58 (0.00, 1.71)
EDE‑Q Eating concern	15	3.28 (1.42)	16	3.63 (1.89)	−0.17 (1.13)	−0.15 (−0.82, 0.48)
EDE‑Q Weight concern	15	4.24 (1.97)	16	3.89 (2.06)	0.37 (1.32)	0.28 (−0.39, 1.14)
EDE‑Q Shape concern	15	4.51 (2.00)	16	4.24 (2.27)	0.40 (1.38)	0.29 (−0.39, 1.20)
EDE‑Q Global	15	3.94 (1.58)	16	3.73 (1.97)	0.36 (1.07)	0.34 (−0.25, 0.98)
RCADS Total Anxiety	17	59.47 (21.37)	16	54.00 (18.91)	6.20 (14.70)	0.71 (−1.34, 19.74)
RCADS Total Anxiety and Depression	17	79.82 (26.91)	16	69.25 (23.24)	9.20 (19.03)	0.48 (−1.34, 19.74)
MR: importance to change	16	3.81 (2.74)	16	5.19 (2.95)	1.27 (2.12)	0.60 (0.09, 2.44)
MR: confidence to change	16	3.88 (3.01)	16	4.50 (3.14)	0.67 (1.99)	0.34 (−0.43, 1.77)
DFlex Cog rigidity	16	54.94 (8.99)	17	53.06 (10.16)	2.13 (7.12)	0.30 (−1.67, 5.92)
DFlex Attention to detail	16	51.25 (9.96)	17	50.00 (8.85)	0.94 (7.07)	0.13 (−2.83, 4.70)
SRS‑2 Total T Score	20	60.35 (10.83)	16	59.63 (12.36)	1.38 (8.22)	0.17 (−3.01, 5.76)

*%EBW* percentage of estimated body weight, *P’s* participants, *d*_*z*_ Standardised mean change with change score standardisation

^a^Change score calculated in the direction of expected improvement (i.e. positive value change score will reflect improvement in this variable at Time 2, and negative change score will reflect worsening of that variable at Time 2)

### Secondary outcomes

We found no significant correlations between the change score in %EBW and the change score on the WCST perseverative errors (r_s_ = 0.05 *p* = 0.83). However, change scores in %EBW were moderately correlated with change scores on the Brixton test (r_s_ = 0.41, *p* = 0.08), and ROCFT (r_s_ = 0.39, *p* = 0.09), although these correlations were not significant.

We found no significant correlations between composite WRAT‑4 score (mean of reading, spelling, and maths subscale scores) and change score on the WCST perseverative errors (r_s_ = 0.16 *p* = 0.50), Brixton test (r_s_ = −0.09 *p* = 0.70), or the ROCFT (r_s_ = 0.16 *p* = 0.51).

#### Correlations between high and low autistic traits and response to group CRT

Table 4 displays differences on the outcome measures between patients scoring high (*n* = 8, 40%) or low (*n* = 12, 60%) on the ASD measures (or with a pre-existing diagnosis of ASD). Low and high scoring ASD groups were based on SCQ and SRS‑2 scores. Standardised mean change with change score standardisation effect size calculations revealed that there was a large effect size improvement on the WCST after group CRT for young people scoring low on the ASD measures (ES = 0.92) compared to a negligible effect size for those scoring high on the ASD measures (ES = 0.18). There was a large effect size improvement on the Brixton test after group CRT for both those scoring low (ES = 1.16) and high (ES = 1.26) on ASD measures. There was a medium effect size improvement in ROCFT scores for young people scoring low on the ASD measures (ES = 0.57) and a large ES improvement for those scoring high on ASD measures (ES = 0.90).

**Table 4:** Evaluating differences in outcome measures between relative low and high scores on ASD measures (or with an ASD diagnosis)

	Time 1			Time 2						
Measures	*N*	Mean	SD	*N*	Mean	SD	Change score^a^ Mean	Change score SD	Effect size *d*_*z*_	95% CI
*Low Scoring ASD*										
WRAT‑4 composite score	12	105.19	29.12	–	–	–	–	–	–	–
WCST perseverative errors (transformed, 1 outlier removed)	11	0.98	0.10	11	0.87	0.13	0.12	0.13	0.92	0.03, 0.20
Brixton test score	12	8.75	3.98	12	5.42	1.83	3.33	2.87	1.16	1.51, 516
ROCFT score	12	1.41	0.43	12	1.58	0.36	0.17	0.30	0.57	−0.03, 0.36
*High Scoring ASD*										
WRAT‑4 composite score	8	95.96	34.17	–	–	–	–	–	–	–
WCST perseverative errors (transformed)	8	0.94	0.25	8	0.91	0.18	0.03	0.17	0.18	−0.12, 0.17
Brixton test score	8	6.88	2.70	8	5.13	2.95	1.75	1.39	1.26	0.59, 2.91
ROCFT score	8	1.34	0.35	8	1.61	0.29	0.27	0.30	0.90	0.02, 0.52

*d*_*z*_ Standardised mean change with change score standardisation

^a^Change score calculated in the direction of expected improvement (i.e. positive value change score will reflect improvement in this variable at Time 2, and negative change score will reflect worsening of that variable at Time 2)

#### Correlations between neuropsychological measures

There was a small to moderate effect size correlation between the two measures of SS (WCST and Brixton test) at Time 1 (*r*_s_ = 0.48, *p* = 0.039) but not at Time 2 (*r*_s_ = 0.42, *p* = 0.074). The change score on the WCST and the change score on the Brixton tests were not significantly correlated (*r*_s_ = 0.02, *p* = 0.94). There was no correlation between the measure of CC (ROCFT) and measures of SS at Time 1, Time 2, or change scores.

#### Protocol adherence and patient feedback

All participants completed the Time 2 assessment following 4 sessions of CRT. Three participants received ‘booster’ individual sessions to help them reach their minimum ‘dose’ due to practical reasons for not making it to the group CRT; of these, 2 participants received their 4th session only as an individual CRT session, and 1 participant received 2 individual sessions. Four participants had received CRT in previous treatment settings, and 2 participants completed up to 4 sessions of CRT prior to their time 1 assessment.

The treatment satisfaction questionnaire revealed that the mean score for treatment satisfaction was 3.35 (SD = 0.86). Of those completing the measure (*n* = 17), the majority of participants (76.5%) rated that they enjoyed the treatment, 5.9% ‘really enjoyed’ it, and 17.6% of participants ‘did not really enjoy it’. The majority (76.5%) rated the sessions as useful, 5.9% rated the sessions as ‘really useful’, and 17.6% rated sessions as ‘not really useful’. All participants completing the measure reported using new thinking skills or strategies as a result of the group, with 29.4% reporting using many or lots of these new thinking skills/strategies, 41.2% using some, and 29.4% using ‘a few’. The majority of participants rated the length of sessions as ‘just right’ (82.4% of participants). Some participants rated the sessions as ‘a bit too short’ (11.8%) or ‘a bit too long’ (5.9%).

Participants left open-ended comments about what they found helpful or less helpful about the group. Ten participants commented on enjoying the games/activities linked to different thinking styles; 6 participants identified enjoying understanding more about their thinking styles and looking at different ways of seeing things; 3 participants commented on enjoying thinking about strategies to practise new thinking styles. Three participants suggested having more examples of how to practise the new skills at home.

## Discussion

The first hypothesis that it would be feasible to recruit a minimum of 20 participants over the planned 10-month recruitment phase was confirmed. Study uptake (96%) and study retention (91%) were also favourable compared to other studies [15]. Study flow was comparable to other studies of CRT summarised in a systematic review showing drop-out rates between 10–15% [11].

The second hypothesis that effect sizes would demonstrate positive change (improvements in SS, CC) was also confirmed using neuropsychological assessment measures. Effect size improvements were similar to individual CRT for young people with AN [15] and adult inpatients with AN [32]. Effect sizes in the current study were larger than previously found for group CRT using the ROCFT and a self-report measure of cognitive flexibility [15] and larger than effect size improvements summarised in a recent systematic review of CRT in AN [14]. Pre-intervention scores on the WCST were similar to those reported in previous similar studies using the WCST and post-intervention scores similar to healthy controls [10]. The pre-intervention scores on the ROCFT in this study were similar to some other studies in young people with AN summarised in a meta-analysis [7]. Academic achievement scores were also similar to those reported in a systematic review [33].

The third hypothesis that the neuropsychological measures employed in the current study sample would be suitable for use in evaluating group CRT (sensitive to change pre and post intervention) was partially confirmed. All three measures demonstrated sensitivity to change pre and post intervention, however, there was an unexpected finding with regards to the two measures of SS. Firstly, a strong ceiling effect was found using the Brixton Test in the current study but not for the WCST (or the ROCFT for CC). At Time 1 (pre-group CRT) 55% of participants achieved a score of 10 which is the maximum possible score on the Brixton Test and this increased at Time 2 to 80% of participants scoring this maximum possible score. Although there was a correlation between the Time 1 score on the Brixton Test and the Time 1 score on the WCST, there was no correlation between the change scores on these measures or the scores at Time 2. One possible interpretation is that these tests were measuring different aspects of SS. However, given that these tests are conceptually alike, another plausible explanation may relate to a methodological issue related to these observed ceiling effects and their impact. Studies in neuropsychological assessment have shown a general test-taking benefit in which enhanced performance may occur after repeated examination, even with different test items [34]. Given the strong ceiling effects on the Brixton Test in the current study, it is likely that greater practise effects occurred using the Brixton Test relative to the WCST. This may have accounted, at least in part, to the larger effect size improvements at Time 2 on the Brixton Test; it is likely that the ‘true’ effect was smaller, and this may explain the lack of correlation between change scores on the Brixton Test and the WCST while the Time 1 score was correlated. Therefore, the results of this study using the Brixton Test must be interpreted with caution. In terms of feasibility and establishing methodology for future larger studies, the WCST is likely to offer a more suitable tool for measuring change in SS in young people with AN following CRT. Another unexpected finding was that less change was found using the self-report measure of SS and CC (the D‑Flex) and this measure did not appear to map onto the experimental measures of SS or CC. The D‑Flex has not yet been validated for use in children and young people with AN and it therefore may not be suitable for use in this group. Another interpretation may relate to increased self-awareness of SS and CC by Time 2 completion of the measure which may produce inflated scores at Time 2 relative to scores at Time 1. This has potential implications for previous studies in which self-report has been used as the main outcome of CRT in the absence of cognitive assessments.

A second aim was to perform exploratory preliminary analysis using the data collected (not outcome analysis as this is a feasibility study) to explore whether improvements in SS and CC related to (i) changes in weight restoration and (ii) scores on ASD measures. Improvements in SS using the Brixton Test, but not the WCST, and improvements in CC were moderately associated with weight restoration. This association was not significant in this study although this may relate to small sample size and low statistical power. Although weight status would be expected to have some degree of impact on cognitive ability [35], Tchanturia and colleagues in an adult AN population [11] and Lang and colleagues in young people [10] have shown that weight and weight gain alone are not predictors of significantly improved cognitive ability.

There were mixed findings with regards to improvements in SS and CC for those scoring high (vs. low) on ASD features; Young people with ASD features showed smaller (using the WCST) and similar (using the Brixton Test) size improvements in SS, but larger sized improvements in CC. This finding was not consistent with a previous study showing smaller effect size improvements in self-report measures of both SS and CC for adults with AN with ASD features (vs. low ASD features) [17]. Furthermore, the first study of CRT in people diagnosed with ASD (without an eating disorder) has recently demonstrated significant improvement in CC and anxiety post-interventions which was maintained at 3‑month follow-up and a large effect size improvement in SS [36]. It has been hypothesized that the atypical executive function profile in ASD may relate more to compensatory strategies over the developmental trajectory, rather than necessarily relating to a ‘deficit’ [37], which adds strength to the notion that skills for SS and CC may be developed. It is therefore plausible that CRT may be beneficial in those with AN with and without ASD features, although further research considering adaptations to maximise acceptability and efficacy for this subgroup in line with treatment recommendations for working with people with ASD as suggested by other studies may be warranted [12, 38]. It is also of note that in this study, while SS and CC demonstrated improvement, the measure of ASD features (the SRS-2) remained unchanged at Time 2 demonstrating the relative robustness of this measure for assessing features of ASD and perhaps less likely exacerbated by other mental health psychopathology [38].

This was an uncontrolled study with group CRT offered as a treatment adjunct, and one inevitable limitation is the extent to which improvements in cognitive functioning were due to the group, other interventions within the day-patient programme, or other uncontrolled variables. Another limitation is that patients included in the analysis completed a different number of group CRT sessions. The study sample also did not include any males; although males were included as per study inclusion criteria, there were no males attending the ITP during the study recruitment period. This study also employed informant-rated measures of ASD features. Additionally, this study does not address the longevity of the observed changes in SS and CC, and future studies may benefit from employing a follow-up period to explore this further. Furthermore, this study was not a classic pre-post design as post evaluation took place after 4 CRT sessions and CRT is designed for 8 sessions, therefore due to this limitation the study uptake or retention may be biased. Internal consistency for the EDE‑Q at Time 2 was poor and therefore this finding must be interpreted with caution. Finally, participants were all receiving treatment within a daypatient programme and therefore likely represent a sub-population of young people with AN receiving treatment. Although illness duration was within the 3 years for the vast majority of young people included in this study (mean was 22 months illness duration), the findings from this study may not generalise to young people with AN in outpatient or inpatient settings who may have milder or more severe illness presentations.

Group CRT was acceptable to the young people and was associated with improvements in cognitive functioning. Important study parameters have been estimated in order to plan a future definitive trial evaluating the effectiveness of group CRT in young people with AN. This study has also shown that group CRT may also be beneficial for young people with AN who present with ASD features.
